# ILD-Slider: A Parameter-Efficient Model for Identifying Progressive Fibrosing Interstitial Lung Disease from Chest CT Slices

**DOI:** 10.3390/jimaging11100353

**Published:** 2025-10-09

**Authors:** Jiahao Zhang, Shoya Wada, Kento Sugimoto, Takayuki Niitsu, Kiyoharu Fukushima, Hiroshi Kida, Bowen Wang, Shozo Konishi, Katsuki Okada, Yuta Nakashima, Toshihiro Takeda

**Affiliations:** 1 D3 Center, The University of Osaka, Osaka 565-0871, Japan; jiahao@is.ids.osaka-u.ac.jp (J.Z.); wang@ids.osaka-u.ac.jp (B.W.); 2 Department of Transformative System for Medical Information, Graduate School of Medicine, The University of Osaka, Osaka 565-0871, Japan; 3 Department of Medical Informatics, Graduate School of Medicine, The University of Osaka, Osaka 565-0871, Japan; sugimoto.kento@hp-info.med.osaka-u.ac.jp (K.S.); konishi.shozo.med@osaka-u.ac.jp (S.K.); katsu-ki@umin.ac.jp (K.O.); ttakeda@hp-info.med.osaka-u.ac.jp (T.T.); 4 Department of Respiratory Medicine, Osaka General Medical Center, Osaka 558-8558, Japan; t.niitsu@imed3.med.osaka-u.ac.jp; 5 Department of Respiratory Medicine and Clinical Immunology, Graduate School of Medicine, The University of Osaka, Osaka 565-0871, Japan; fukushima@imed3.med.osaka-u.ac.jp; 6 Department of Respiratory Medicine, National Hospital Organization, Osaka Toneyama Medical Center, Osaka 560-8552, Japan; kida.hiroshi.sv@mail.hosp.go.jp; 7 SANKEN, The University of Osaka, Osaka 567-0047, Japan; n-yuta@im.sanken.osaka-u.ac.jp

**Keywords:** PF-ILD identification, parameter-efficient transfer learning, medical image analysis, interstitial lung disease, chest CT, deep learning

## Abstract

Progressive Fibrosing Interstitial Lung Disease (PF-ILD) is a severe phenotype of Interstitial Lung Disease (ILD) with a poor prognosis, typically requiring prolonged clinical observation and multiple CT examinations for diagnosis. Such requirements delay early detection and treatment initiation. To enable earlier identification of PF-ILD, we propose ILD-Slider, a parameter-efficient and lightweight deep learning framework that enables accurate PF-ILD identification from a limited number of CT slices. ILD-Slider introduces anatomy-based position markers (PMs) to guide the selection of representative slices (RSs). A PM extractor, trained via a multi-class classification model, achieves high PM detection accuracy despite severe class imbalance by leveraging a peak slice mining (PSM)-based strategy. Using the PM extractor, we automatically select three, five, or nine RSs per case, substantially reducing computational cost while maintaining diagnostic accuracy. The selected RSs are then processed by a slice-level 3D Adapter (Slider) for PF-ILD identification. Experiments on 613 cases from The University of Osaka Hospital (UOH) and the National Hospital Organization Osaka Toneyama Medical Center (OTMC) demonstrate the effectiveness of ILD-Slider, achieving an AUPRC of 0.790 (AUROC 0.847) using only five automatically extracted RSs. ILD-Slider further validates the feasibility of diagnosing PF-ILD from non-contiguous slices, which is particularly valuable for real-world and public datasets where contiguous volumes are often unavailable. These results highlight ILD-Slider as a practical and efficient solution for early PF-ILD identification.

## 1. Introduction

Interstitial Lung Disease (ILD) is a chronic condition characterized by lung infiltration and fibrosis, ultimately leading to respiratory failure. Early diagnosis is crucial for improving patient survival; however, it remains challenging due to the heterogeneous clinical presentations of ILD [1]. Although lung biopsy is considered the gold standard for diagnosis, it is not always feasible because of patient-related factors such as contraindications, patient preference, or an inability to undergo the procedure. Moreover, CT examinations over a defined observation period are often required to assess fibrotic progression, inevitably delaying both diagnosis and treatment initiation.

Diagnosing ILD demands the expertise of specialized pulmonologists, radiologists, and pathologists, which may not always be readily available. Accurate diagnosis is difficult, and predicting disease progression is even more challenging due to variability among patients. One subtype, Progressive Fibrosing Interstitial Lung Disease (PF-ILD), carries a poor prognosis, with a median survival of only 3–5 years after diagnosis [2,3,4]. Recently, the antifibrotic drugs nintedanib and pirfenidone have been shown to slow fibrosis and extend survival [5,6], making timely and accurate PF-ILD identification essential for initiating treatment. This underscores the need for reliable early diagnostic methods.

Deep learning has led to significant advances across multiple domains, including autonomous driving [7], computer-aided diagnosis [8,9,10], and smart cities [11,12]. In computer vision, the emergence of vision foundation models [13,14,15] has enabled high performance in large-scale, complex tasks. Medical image analysis has similarly benefited, achieving remarkable diagnostic accuracy using image data alone [16,17] or in combination with textual information [18]. These advances have inspired the development of frameworks to support PF-ILD identification. Typically, identification relies on high-resolution CT (HRCT) scans, which provide 3D volumetric data. However, applying 3D models to PF-ILD identification poses two main challenges: (1) large annotated datasets are scarce and (2) 3D models involve a vast number of trainable parameters, demanding substantial computational resources.

These challenges highlight the urgent need for data-efficient and lightweight diagnostic frameworks. Parameter-efficient transfer learning (PETL) has gained attention in natural language processing (NLP) for adapting large models to specific downstream tasks with minimal parameter updates. Methods such as task-specific adapters [19,20,21] and prompt tuning [22,23,24,25] introduce small learnable modules or tokens, substantially reducing the number of trainable parameters. PETL approaches have also been explored in computer vision, including prefix tuning [26,27], adapters [28,29], and prompt tuning [30,31,32,33].

Building on the success of PETL methods [19,28], we propose ILD-Slider, a parameter-efficient, lightweight framework for PF-ILD identification. ILD-Slider integrates a slice-level 3D Adapter (Slider) with a peak slice mining (PSM)-based position marker (PM) extractor to identify representative slices (RSs). At the case level, we define anatomy-based PMs to guide RS selection, as processing all slices is computationally prohibitive. PMs are manually defined as distinct classes and used to train a multi-class classification model. Our PSM-based strategy then selects key slices based on PM indices, from which three, five, or nine RSs are extracted. Finally, these RSs are used to train the Slider for efficient and accurate PF-ILD identification.

The contributions of this paper are as follows:

- We introduce anatomy-based PMs to guide the selection of RSs for PF-ILD identification. A PM extractor, trained with a standard multi-class classification model, achieves high extraction accuracy despite severe class imbalance. This enables the use of a small set of RSs instead of full CT volumes, thereby improving diagnostic efficiency and significantly reducing computational cost.
- We design a PSM-based strategy to automatically select RSs from PM indices. Using datasets from two medical facilities, ILD-Slider achieves an AUPRC of 0.790 and an AUROC of 0.847 with only five extracted RSs. We also propose an effective window level/width processing for PF-ILD identification, validate its effectiveness, and analyze domain shifts on different facilities, demonstrating the robustness of our Slider.
- We demonstrate the feasibility of using non-contiguous slices for PF-ILD identification. This is particularly valuable for real-world and public datasets, where contiguous slices are often unavailable, underscoring the practicality and generalizability of ILD-Slider.

## 2. Related Work

### 2.1. Medical Image Classification

Recently, significant progress has been made in significant progress in applying convolutional neural networks (CNNs) to medical image classification [34], greatly facilitated by the availability of open-source 2D pre-trained models for related medical tasks has greatly facilitated these advancements. Such models, typically pre-trained on large-scale natural image datasets (e.g., ImageNet [35]), can be fine-tuned for various downstream medical applications. Although medical datasets are generally more difficult to obtain than natural image datasets, pre-trained weights have proven to be highly beneficial in medical imaging. Li et al. [36] proposed a shallow convolutional architecture for classifying ILD in lung image patches. Sakamoto et al. [37] introduced a novel cooperative deep learning method for classifying diverse pathology-based medical images. Nithiyaraj et al. [38] developed a CT slice classification model to assist radiologists in selecting diagnostically valuable slices. Due to the limited availability and imbalance of medical data, weakly supervised, unsupervised, and self-supervised learning approaches have emerged as promising research directions. For example, Li et al. [39] proposed a multi-scale convolutional model that utilizes a shared set of convolutional kernels to extract features with different receptive fields, achieving strong performance in comparison to medical image classification benchmarks.

Inspired by these works, we adopt a conventional CNN model to extract representative slices from each case and address the extreme class imbalance through both oversampling and downsampling. Furthermore, we demonstrate that the proposed method can effectively select a small set of informative slices for PF-ILD identification.

### 2.2. Parameter-Efficient Transfer Learning (PETL)

In NLP, prompting is a widely used technique for guiding large language models (LLMs) to better adapt to downstream tasks [40]. For instance, in-context learning (ICL) [41,42,43,44,45] and visual ICL [32,46,47,48,49] demonstrates remarkable generalization across diverse tasks via, but often relies on carefully crafted, manually designed prompts. However, fully fine-tuning large-scale models is computationally expensive due to the vast number of parameters involved. To address this issue, parameter-efficient transfer learning (PETL) methods have been proposed. These approaches optimize only a small subset of the model’s parameters or introduce additional lightweight modules, such as adapters [19,50] and prompt tuning [51,52], achieving performance comparable to full fine-tuning while substantially reducing computational cost.

Building on the success of PETL in NLP, recent research has extended these techniques to computer vision [30,31] and vision–language modeling [14,26,27]. In these domains, PETL typically involves either partial fine-tuning of the backbone model or the insertion of learnable prompts into image inputs. For example, Pan et al. [28] enhanced a traditional adapter [19] with a 3D convolutional layer, enabling the Vision Transformer (ViT) [53] to effectively carry out video action recognition tasks. This method updates only a small fraction of the model’s parameters, making adapters an attractive solution for computationally constrained medical applications such as CT slice analysis.

Inspired by these developments, we explore adapter-based PETL methods to improve performance in medical imaging tasks involving CT scans.

### 2.3. Prognostic Prediction for ILD

Furukawa et al. [54] developed a classification approach for idiopathic pulmonary fibrosis (IPF), a major subtype of PF-ILD with a poor prognosis. They employed a semantic segmentation model to delineate fibrotic regions in the lungs and compared the results with non-invasive measures using a diagnostic algorithm for IPF. Ryerson et al. [55] introduced the GAP model to predict mortality risk in chronic ILD. The ILD-GAP model demonstrated robust performance in estimating mortality rates across major subtypes of chronic ILD and at all disease stages. Walsh et al. [56] evaluated the performance of a widely used deep learning model for diagnosing fibrotic diseases—the systematic Objective Fibrotic Imaging Analysis Algorithm (SOFIA)—in predicting usual interstitial pneumonia and found that it outperformed radiologists in identifying progressive fibrotic lung disease.

To the best of our knowledge, only a few published studies have explored machine learning-based approaches for PF-ILD identification. We are the first to propose the use of representative slices for PF-ILD identification and demonstrate promising results.

## 3. Method

### 3.1. Study Population

We collected chest CT scans from patients diagnosed with ILD at The University of Osaka Hospital (UOH) and the National Hospital Organization Osaka Toneyama Medical Center (OTMC). In this study, we utilized a dataset that was previously established in our prior work [57]. Patient inclusion and the definition of PF-ILD followed the same criteria as described therein, namely ≥10% fibrosis on HRCT and a relative decline of either ≥ 10% or >5% to <10% in forced vital capacity (FVC), accompanied by clinical deterioration or radiological progression during overlapping two-year windows, with baseline ≥ 45%. Initially, we identified 1126 ILD cases (UOH, 462; OTMC, 664), which was reduced to 1049 after excluding cases with inadequate CT image quality or incomplete lung coverage (UOH, 28; OTMC, 49). Of these, we further excluded cases missing labels that were required to ascertain PF-ILD status (UOH, 31; OTMC, 405), leaving 613 cases for PF-ILD identification.

### 3.2. Overview

Let $S=\{x_{1},x_{2},\ldots ,x_{N}\}$ denote the dataset used for PF-ILD identification, consisting of *N* cases. Each case is represented as $x_{i}=\left\{x_{i}^{1},x_{i}^{2},\ldots ,x_{i}^{M}\right\}$, where *M* denotes the number of CT slices. We employ a classification model $S_{\theta }$, together with a PSM module to extract PMs. RSs are then selected based on the detected PMs and subsequently fed into the proposed diagnostic framework.

**Figure 1:**
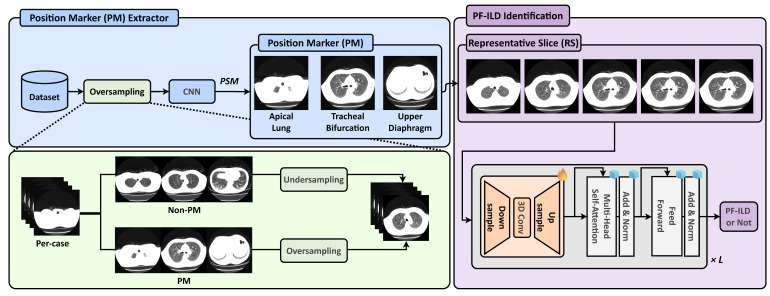
Overview of the proposed ILD-Slider framework. A PM extractor and PSM are used to identify the predefined PMs: apical lung, tracheal bifurcation, and upper diaphragm. To address severe class imbalance, the PM extractor is trained on a class-balanced dataset constructed by oversampling the PM classes and undersampling the Non-PM class. Based on the extracted PMs, RSs are selected for subsequent PF-ILD identification. Finally, a lightweight slice-level 3D Adapter (Slider), equipped with tunable adapters in each transformer block, performs the final PF-ILD identification.

As illustrated in Figure 1, we define three types of PMs: apical lung, tracheal bifurcation, and upper diaphragm. A CNN-based PM extractor is trained to identify these PMs, while the PSM module is applied during inference to select the most relevant slices. To address the severe inter-class imbalance during training, we adopt an oversampling strategy for the PM classes and downsample the non-PM class to balance the dataset. After PM extraction, the RSs corresponding to these PMs are selected and input into a vision foundation model (DINOv2 [13]) with a frozen backbone. A slice-level 3D Adapter (Slider) equipped with trainable adapters is inserted into each transformer block, enabling parameter-efficient fine-tuning for downstream PF-ILD identification.

### 3.3. Position Marker Selector

#### 3.3.1. PM Extractor Training

In our dataset, the CT scans have a default slice interval of 4 mm, resulting in approximately 75 slices per case. We define and label three PMs to represent distinct anatomical landmarks in the lungs:

The apical lung (upper) PM. The slice where aerated lung parenchyma first appears in either hemithorax is labeled as the upper PM. During the operation, (i) the air in the trachea is ignored, (ii) when both the current slice and the next caudal slice show early parenchyma, the more superior slice is selected, and (iii) at asymmetric onset, the earlier-appearing hemithorax is used.

The tracheal bifurcation (middle) PM. The slice where the trachea first begins to bifurcate into the right and left main bronchi. A slice is considered the middle PM if all the following criteria hold: (i) when the next caudal slice displays unequivocal bifurcation but the previous superior slice does not, the current slice is selected as the middle PM (the first-bifurcation level), and (ii) two distinct bronchial lumens are present and separated by a soft-tissue wall (no longer a single circular tracheal lumen).

The upper diaphragm (lower) PM. The lower PM is defined as the axial slice located three to four slices superior to the level where aerated lung parenchyma completely disappears bilaterally. We determine the disappearance level using the following operational criteria: (i) the next caudal slice shows no aerated lung parenchyma in both hemithoraces, whereas the previous superior slice still shows aerated parenchyma; (ii) if one lung disappears earlier, we continue scrolling caudally until both lungs are absent; (iii) we ignore any low-attenuation regions that are not aerated lung parenchyma.

The labeling procedure is rule-based and reproducible without requiring radiological expertise. For quality assurance, an experienced physician reviewed and approved the final set of annotations, confirming the anatomical validity of the definitions.

To train a PM extractor, we construct a classification dataset $S_{\mathrm{PM}}$, in which each slice is assigned a PM class label. Since only one slice per case belongs to each PM class, the dataset exhibits severe imbalance between PM and non-PM categories. To address this issue, we apply a combination of oversampling and downsampling: specifically, non-PM slices are randomly downsampled by 75%, while PM slices are oversampled to match the number of non-PM samples. Furthermore, we adopt a weighted loss function to penalize false positives and further mitigate the effects of class imbalance.

#### 3.3.2. Peak Slice Mining (PSM)

After training, we apply a case-level PSM function, *r*, to select one slice per PM class for each case. Given a case, $x_{n}\in S$, with *M* slices, the PM extractor, $S_{\theta }$, outputs a confidence tensor, $c_{n}\in R^{M\times C}$, where *C* is the number of PM classes.

To obtain a normalized confidence distribution over slices for each class, we apply the softmax function, σ(·), along the slice dimension *M*, independently for each class. For every PM class *c*, this ensures that the probabilities across all *M* slices in the case sum to 1. Formally, we can express this as follows:

(1) $$\begin{array}{cc} \sigma {\left(c_{n}\right)}_{m,c} & =\frac{\exp(c_{n,m,c})}{\sum_{m^{\prime }=1}^{M}\exp\left(c_{n,m^{\prime },c}\right)}, \end{array}$$

(2) $$\begin{array}{cc} r_{c} & =\arg\max_{m}\sigma {\left(c_{n}\right)}_{m,c},\forall c\in \{1,\ldots ,C\}. \end{array}$$

where $\sigma {\left(c_{n}\right)}_{m,c}$ denotes the normalized probability that the *m*-th slice in case $x_{n}$ corresponds to PM class *c*. For each c∈{1,…,C}, we select the slice index $r_{c}$ with the highest probability as the representative PM slice. Formally, the PSM function *r* maps the confidence tensor $c_{n}$ to the set of selected indices:

(3) $$\begin{array}{c} R=r\left(c_{n}\right)=\left\{r_{1},r_{2},\ldots ,r_{C}\right\}. \end{array}$$

The set, *R*, thus contains one peak slice for each PM class, which is subsequently input into the downstream PF-ILD identification task.

#### 3.3.3. Representative Slice (RS) Extraction

**Figure 2:**
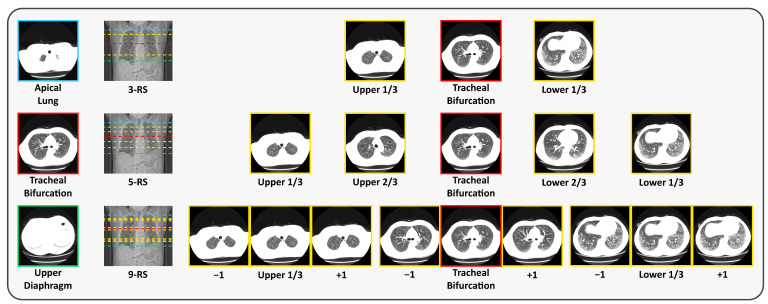
Visual examples of the position markers (PMs) and representative slices (RSs). The locations of the PMs and RSs are indicated on the longitudinal CT view, with the corresponding axial CT slices shown alongside (best viewed in color). The PMs include apical lung (upper), tracheal bifurcation (middle), and upper diaphragm (lower). The RSs are shown from top to bottom as 3-RS, 5-RS, and 9-RS. Here, −1 and +1 denote the adjacent slices superior and inferior to the current slice, respectively.

During preprocessing, DICOM files are converted into PNG format, and slices within each case are renamed sequentially from the apical lung to the diaphragm. This conversion facilitates RS extraction based on the predefined PMs. We adopt three RS extraction strategies, using three, five, or nine RSs, as shown in Figure 2. For the 3-RS strategy, the upper RS is defined as the slice located at one-third of the distance between the apical lung PM and the tracheal bifurcation PM; the middle RS is the tracheal bifurcation PM itself; and the lower RS is the slice located at one-third of the distance between the tracheal bifurcation PM and the upper diaphragm PM. For the 5-RS strategy, we select slices at one-third and two-thirds of the interval between the apical lung PM and the tracheal bifurcation PM, the tracheal bifurcation PM itself, and two-thirds and one-third of the interval between the tracheal bifurcation PM and the upper diaphragm PM. For the 9-RS strategy, we extend the 3-RS approach by including the immediate neighboring slices before and after each of the three selected RSs, resulting in nine slices in total.

By anchoring RS extraction to PMs, we eliminate the need to account for inter-patient variations in lung size and morphology. This approach allows the model to focus on consistent, anatomically defined features, thereby standardizing RS selection across all cases.

### 3.4. PF-ILD Identification

#### 3.4.1. Preliminaries

Adapters [19] were originally introduced as a PETL technique in NLP. An adapter module typically consists of a down-projection linear layer followed by an up-projection linear layer. Given an input feature matrix, $X\in R^{L\times d}$, at the *i*-th layer, the adapter transformation can be formulated as follows:

(4) $$\begin{array}{c} \mathrm{Adapter}\left(X\right)=X+f\left(W_{\mathrm{down}}X\right)W_{\mathrm{up}}, \end{array}$$

where $W_{\mathrm{down}}\in R^{d\times p}$ is the down-projection weight matrix, $W_{\mathrm{up}}\in R^{p\times d}$ is the up-projection weight matrix, and f(·) denotes a non-linear activation function. The bottleneck dimension *p* is typically defined as $p=\frac{d}{\delta }$, where δ is the reduction factor.

Adapters have shown strong performance in NLP tasks, as they only introduce a small number of task-specific trainable parameters while keeping the backbone model frozen. This not only reduces memory usage and computational cost but also mitigates catastrophic forgetting, a common issue with full fine-tuning [19].

#### 3.4.2. Slice-Level 3D Adapter (Slider)

A conventional adapter is limited to 2D feature representations and only performs spatial modeling across tokens. However, CT scans are inherently 3D, and in PF-ILD identification, the spatial arrangement of slices along the axial dimension (superior to inferior) encodes critical anatomical and pathological progression. Inspired by the ST-Adapter [28], we extend the standard adapter by introducing a slice-level 3D Adapter (Slider) to efficiently capture inter-slice dependencies. Similarly to the original adapter, our Slider includes a down-projection layer, a non-linear activation layer, and an up-projection layer. Between these layers, we insert a depth-wise separable 3D convolution layer to perform slice-level reasoning. Formally, the Slider transformation is defined as follows:

(5) $$\begin{array}{c} 3D-\mathrm{Adapter}\left(X\right)=X+f\left(3\mathrm{DConv}\left(W_{\mathrm{down}}X\right)\right)W_{\mathrm{up}}, \end{array}$$

where $X\in R^{T\times L\times d}$ denotes the patch features from *T* CT slices, f(·) is a non-linear activation function, and 3DConv is a depth-wise 3D convolution operating across the slice dimension *T* and the spatial patch grid. As in the conventional adapter, $W_{\mathrm{down}}\in R^{d\times p}$ and $W_{\mathrm{up}}\in R^{p\times d}$ are the down-projection and up-projection weight matrices, respectively. The bottleneck dimension *p* is defined as $p=\frac{d}{\delta }$, where δ is the reduction factor. Before applying 3DConv, the down-projected features are reshaped from sequence format to volumetric form, i.e., $X^{\prime }\in R^{T\times L\times d}\to X^{\prime \prime }\in R^{T\times H\times W\times d}$, where *H* and *W* denote the patch grid height and width, respectively. This reshaping enables the 3D convolution to jointly model spatial and slice-level contexts.

By inserting this adapter module into each transformer block of a frozen ViT backbone, the model can extract richer 3D representations without modifying the pre-trained weights. In PF-ILD identification, this allows the model to capture disease-related structural patterns that evolve across lung slices, thereby enhancing PF-ILD identification.

#### 3.4.3. Preprocessing

**Figure 3:**
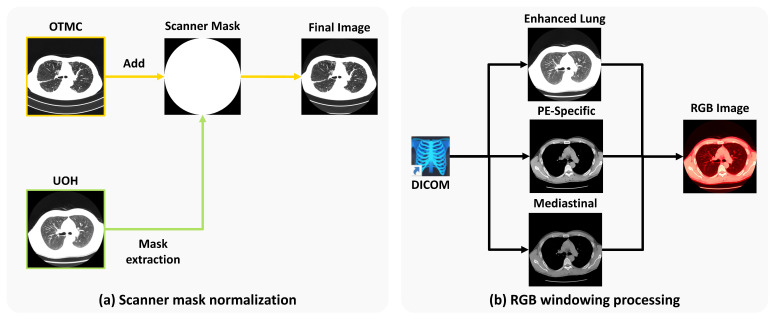
(**a**) Scanner mask normalization: We extract the scanner mask from UOH and use it on data from OTMC to mitigate the disparity across the two facilities. (**b**) RGB windowing processing: We perform three different types of organ-specific windowing and concatenate them into an RGB image. “PE” stands for pulmonary embolism.

Scanner mask normalization. In this study, we utilize datasets collected from two different medical facilities. Due to variations in diagnostic equipment, such as differences in CT scanner models (see Figure 3a), we observe inconsistencies in non-anatomical regions across scans. To address this issue, we apply a unified scanner mask to both the OTMC and UOH datasets. Specifically, we generate a binary scanner mask from the UOH dataset and subsequently apply it to the OTMC dataset. The UOH dataset is also processed using the same standardized mask to ensure consistency across both datasets.

RGB windowing processing. To enhance visual features that are relevant to PF-ILD, we perform a multi-window image fusion process, starting with the original DICOM scans (see Figure 3b). Windowing is performed in Hounsfield units (HU), and each DICOM image is converted into three separate single-channel window images:

- Enhanced lung window: Level = −700 HU; width = 700 HU;
- Pulmonary embolism (PE)-specific window: Level = 100 HU; width = 700 HU;
- Mediastinal window: Level = 40 HU; width = 400 HU.

Each windowed image is resized to 518×518 and then stacked to form a three-channel image of size 518×518×3, analogous to an RGB image, for compatibility with the DINOv2 pre-trained backbone. This transformation enhances the visibility of fibrotic regions in the lung fields within the PNG outputs, thereby improving the model’s ability to accurately identify PF-ILD cases.

## 4. Experiments

### 4.1. Experimental Setup

#### 4.1.1. Datasets

We performed a basic screening process on our datasets, confirming that each patient contributed only one case and ensuring that there was no data leakage between the training and test sets. After screening, 434 cases from the UOH and 615 cases from the OTMC remained, totaling 1049 CT scans. Among these, 403 cases from the UOH and the 210 cases from OTMC were retained with PF-ILD labels, as shown in Table 1.

PM extractor dataset: We randomly select 200 cases from each facility (400 cases in total) from the 1049 available cases, regardless of PF-ILD status. All images are converted from the original DICOM format to 8-bit PNG format based on HU, using a Level of −700 HU and a Width of 700 HU, followed by scanner mask normalization. The dataset is split into 70%, 15%, and 15% for training, validation, and testing, respectively.

PF-ILD identification dataset: We use all available cases with PF-ILD labels for identification, totaling 613 cases (403 from the UOH and 210 from the OTMC). These cases undergo scanner mask normalization and RGB windowing processing to better highlight fibrotic regions based on clinical knowledge. The dataset is also divided into 70%, 15%, and 15% for training, validation, and testing, respectively.

#### 4.1.2. Implementation Details

All PM extractor and Slider modules are implemented in the PyTorch framework (version 1.13.1) and trained on servers equipped with two NVIDIA RTX 6000 Ada Generation GPUs. For the PM extractor, we use EfficientNet-b4 [58] as the classification backbone. Input images are resized to 256×256 and normalized to the intensity range [0,1]. Models are trained for 20 epochs with a batch size of 256 using weighted cross-entropy loss and the Adam optimizer [59], with an initial learning rate of $1\times 10^{-3}$. Data augmentation is applied during training. For the Slider, we employ DINOv2 [13] as the vision foundation model backbone, with the adapter dimension *d* set to 192, corresponding to a reduction factor δ=4. Input images are resized to 518×518. Models are trained for 80 epochs with a batch size of 8 using class-weighted cross-entropy loss (weight ratio 1:1.5), scanner mask normalization, and RGB windowing processing. Optimization is performed using the Adam optimizer, with an initial learning rate of $2\times 10^{-4}$, a cosine annealing learning rate scheduler, and a dropout rate of 0.3.

#### 4.1.3. Evaluation Metrics

**1-Up-Down Accuracy.** To evaluate the performance of the PM extractor, we adopt a relaxed evaluation criterion called 1-Up-Down Accuracy. In clinical practice, the slice immediately above or below the ground-truth PM often contains similar anatomical features. Therefore, we consider a prediction correct if the predicted slice index, $\hat{m}$, satisfies the following inequality:

(6) $$\begin{array}{c} |\hat{m}-m^{*}|\leq 1, \end{array}$$

where $m^{*}$ denotes the ground-truth PM slice index. The 1-Up-Down Accuracy is then defined as follows:

(7) $$\begin{array}{c} 1-\mathrm{Up}-\mathrm{Down}\mathrm{Accuracy}=\frac{1}{N}\sum_{i=1}^{N}1\left(|{\hat{m}}_{i}-m_{i}^{*}|\leq 1\right), \end{array}$$

where *N* is the number of evaluated cases and 1(·) is the indicator function, which returns 1 if the condition is true and 0 otherwise.

For the PF-ILD identification task, we adopt several metrics for evaluation:

**AUROC.** The Area Under the Receiver Operating Characteristic Curve (AUROC) is widely used for binary classification and measures the model’s ability to distinguish between healthy and diseased samples across various classification thresholds. Given predicted scores, ${\hat{y}}_{i}$, and true labels, $y_{i}\in \left\{0,1\right\}$, for i=1,…,N, the AUROC is defined as follows:

(8) $$\begin{array}{c} \mathrm{AUROC}=\frac{1}{N_{+}N_{-}}\sum_{i:y_{i}=1}\sum_{j:y_{j}=0}1\left({\hat{y}}_{i}>{\hat{y}}_{j}\right), \end{array}$$

where $N_{+}$ and $N_{-}$ are the number of healthy and diseased samples, respectively.

The metrics below are calculated from the following components of the confusion matrix at a specific threshold of 0.5: True Positives (TPs), True Negatives (TNs), False Positives (FPs), and False Negatives (FNs).

**Accuracy (Acc.)** measures the proportion of all samples that are correctly classified:

(9) $$\begin{array}{c} \mathrm{Accuracy}=\frac{TP+TN}{TP+TN+FP+FN} \end{array}$$

**Recall (Rec.)** measures the proportion of actual positive samples that are correctly identified and is crucial for minimizing missed diagnoses:

(10) $$\begin{array}{c} \mathrm{Recall}=\frac{TP}{TP+FN} \end{array}$$

**Precision (Prec.)** measures the proportion of positive predictions that are correct, indicating the reliability of a positive diagnosis:

(11) $$\begin{array}{c} \mathrm{Precision}=\frac{TP}{TP+FP} \end{array}$$

**Specificity (Spec.)** measures the proportion of actual negative samples that are correctly identified, reflecting the model’s ability to rule out the condition:

(12) $$\begin{array}{c} \mathrm{Specificity}=\frac{TN}{TN+FP} \end{array}$$

**F1-Score (F1)** is the harmonic mean of precision and recall, providing a balanced measure of a model’s performance, which is especially useful in cases of class imbalance:

(13) $$\begin{array}{c} F1-\mathrm{Score}=2\cdot \frac{\mathrm{Precision}\cdot \mathrm{Recall}}{\mathrm{Precision}+\mathrm{Recall}}=\frac{2TP}{2TP+FP+FN} \end{array}$$

**AUPRC.** The Area Under the Precision–Recall Curve (AUPRC) is another threshold-independent metric. It summarizes the trade-off between precision and recall across all possible thresholds. The AUPRC is particularly informative for imbalanced datasets, as it focuses on the performance of the minority (positive) class and is less influenced by the large number of true negatives than the AUROC.

**Statistical comparison.** To formally compare the models using each metric, we estimated 95% confidence intervals for the between-model difference ($\Delta ={\mathrm{metric}}_{\mathrm{Slider}}-{\mathrm{metric}}_{\mathrm{baseline}}$) using a class-stratified, paired bootstrap at the patient level (B=5000 resamples; the same resampled indices were applied to both models). Two-sided *p*-values were obtained via a within-case score-swapping permutation test for the AUPRC and DeLong’s test for the AUROC. All tests were two-sided with α=0.05.

#### 4.1.4. Comparison Methods

For the PM extractor, we evaluate several model families, including ResNet [60], DenseNet [61], and EfficientNet [58]. For the Slider, we compare our method against several transfer learning baseline methods:

- **Full fine-tuning:** fully updates all parameters of the backbone for PF-ILD identification.
- **Partial fine-tuning:** updates only the last ViT layer while keeping all other layers frozen.
- **Linear probe:** trains only the linear classification layer, keeping all other parameters fixed.

### 4.2. Results of PSM-Based PM Extractor

We evaluate the performance of various backbone models for the PSM-based PM extractor on the same training and testing datasets using the 1-Up-Down Accuracy metric. The models span several architecture families, including ResNet [60], DenseNet [61], and EfficientNet [58], and the best-performing model from each family is summarized in Table 2.

Among all the models, EfficientNet-b4 achieves the highest overall performance, with an average 1-Up-Down Accuracy of 98.33%. Its class-wise performance is also strong, reaching 100% for the upper PM, 98.33% for the middle PM, and 96.67% for the lower PM extraction. Within the DenseNet family, DenseNet-169 performs the best, achieving an average 1-Up-Down Accuracy of 97.78%. Its accuracy for the upper and middle PM classes is comparable to that of EfficientNet-b4. These results suggest that the upper PM is a relatively easy prediction target, likely because it corresponds to the slice just before the lung fields become visible, a visually distinct and consistent anatomical feature. In contrast, identifying the lower PM is more challenging as there is greater variability in lung morphology near the diaphragm across patients.

**Figure 4:**
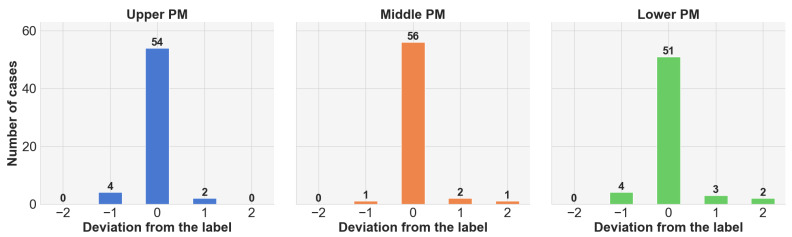
Statistics on the extracted slices from the PSM-based PM extractor on EfficientNet-b4.

To further validate the effectiveness of the PSM-based PM extractor, we analyze the deviation of predicted slices from ground-truth PMs using EfficientNet-b4, as shown in Figure 4. We find that the majority of predictions are either exactly correct or within one slice of the ground truth. Notably, even incorrect predictions remain within two slices above or below the reference PM, demonstrating the robustness and reliability of the proposed PSM-based approach.

### 4.3. Results on Slider for PF-ILD Identification

Table 3 summarizes the PF-ILD identification performance of the proposed Slider model under three different RS configurations. In the 5-RS setting, Slider achieves the following results: AUPRC=0.790 (95% CI [0.652, 0.901]) and AUROC=0.847 (95% CI [0.760, 0.921]). This corresponds to improvements of +0.008 and +0.033 over full fine-tuning (AUPRC=0.782, 95% CI [0.645, 0.894]; AUROC=0.814, 95% CI [0.706, 0.907]). As detailed in the Methods (statistical comparison) section, we compared the AUROC using DeLong’s test, yielding the following result: ΔAUROC=0.033 (95% CI [−0.049, 0.115]; z=0.783; p=0.434). We compared the AUPRC using a class-stratified, paired bootstrap of the difference (Δ) to obtain 95% CIs, with ΔAUPRC = 0.008 (95% CI [−0.073, 0.086]), and computed a within-case score-swapping permutation *p*-value (p=0.854). All tests were two-sided at the α=0.05 significance level. Overall, the point estimates favor Slider, but the differences are not statistically significant; notably, Slider demonstrates comparable performance with substantially fewer trainable parameters (3.56 M), signifying its computational efficiency and deployability.

Across all configurations, 5-RS yields the best overall performance, followed by 9-RS and then 3-RS. Notably, in the 5-RS setting, Slider outperforms full fine-tuning on nearly all metrics except recall, highlighting its strong parameter efficiency and effectiveness. Partial fine-tuning achieves the second-highest AUROC (0.832), confirming that lightweight adaptation remains competitive. Linear probe achieves relatively high recall (0.730) but suffers from low precision and specificity, resulting in a lower AUPRC (0.724) and AUROC (0.774). These results demonstrate that Slider achieves the best trade-off between model complexity and diagnostic accuracy, making it particularly well suited for realistic clinical deployment.

### 4.4. Domain Shift Analysis

In practical applications, domain shifts frequently occur, representing variations between the training dataset, S, and the target environment. These discrepancies reduce performance when models are applied beyond their original training distribution. Such shifts are prevalent across datasets and are widely used as benchmarks to evaluate the robustness of machine learning models [62]. To investigate ILD-Slider’s resilience to domain shifts, we train Slider models on datasets from different facilities and evaluate them using the AUROC with δ=4. The results are summarized in Table 4.

In general, datasets from different facilities exhibit distinct data regimes. The OTMC is less heterogeneous, so Slider can learn it more easily, while the UOH dataset is more heterogeneous. Models trained on a single facility show strong in-domain performance but suffer from notable degradation when tested on data from a different facility. For example, the Slider trained on the UOH dataset achieves an AUROC of 0.750 on this data, but the AUROC increases to 0.896 on OTMC data, whereas the model trained on the OTMC dataset attains 0.921 on this data, yet the value drops to 0.759 on UOH data. In contrast, the model trained on both the UOH and OTMC datasets demonstrates the most balanced and robust behavior, achieving AUROCs of 0.823 on UOH data, 0.875 on OTMC data, and 0.847 on the combined UOH and OTMC test set. These findings underscore the importance of multi-facility training for Slider, which is critical for reliable PF-ILD identification across different scanners and institutions.

### 4.5. Visualization of TPs, TNs, FPs, and FNs

**Figure 5:**
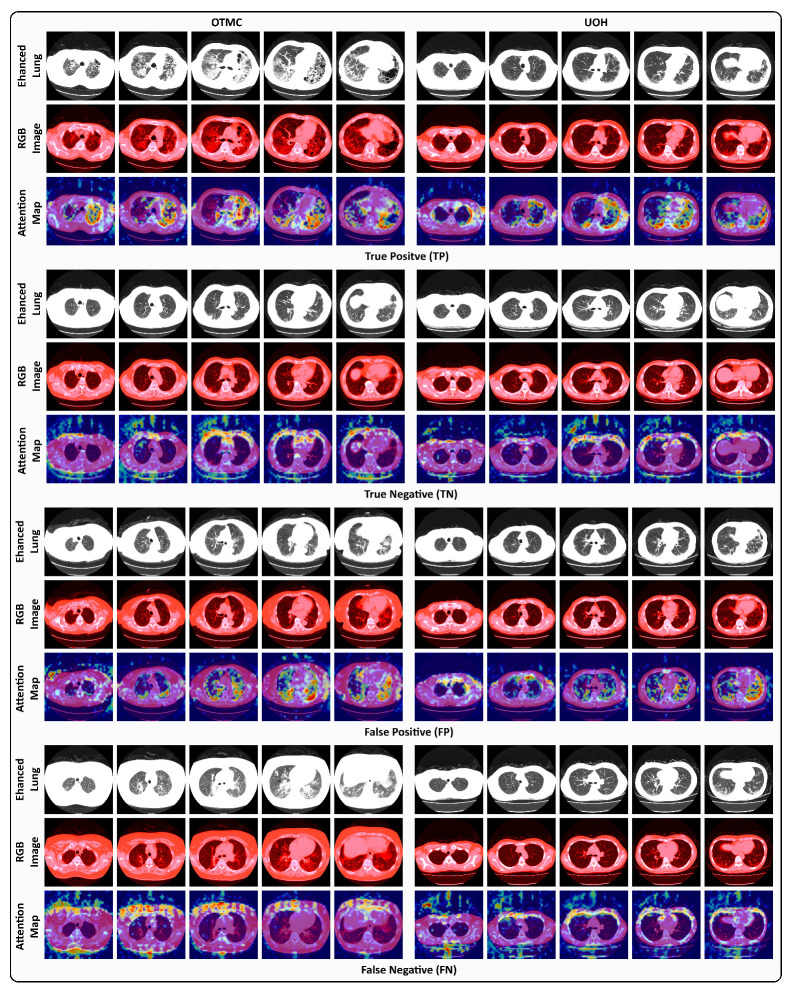
Visual examples under the 5-RS setting of TP, TN, FP, and FN cases from the OTMC and UOH datasets. Each case presents the enhanced lung window image (Enhanced Lung), the RGB windowing-processed image (RGB Image), and the Grad-CAM attention map (Attention Map) corresponding to the predicted class.

To better understand the behavior of the proposed model in PF-ILD identification, representative TP, TN, FP, and FN examples are shown in Figure 5, using Grad-CAM [63] to highlight attention maps for the predicted class. For TP cases, Slider effectively captures fibrosis-related features across slices, enabling accurate PF-ILD identification. For TN cases, the model predominantly focuses on non-lung regions, consistent with the absence of representative fibrosis in lung tissue. In FP cases, however, Slider incorrectly highlights fibrotic-like patterns within the lung field; although fibrosis is present, it is insufficient to confirm PF-ILD, leading to misclassification. These instances illustrate the clinical difficulty of distinguishing incidental fibrosis from PF-ILD. FN cases further reveal model limitations. For OTMC data, Slider mistakenly attends to non-lung regions (e.g., surrounding tissues) while overlooking fibrosis within the lungs. Similarly, for UOH data, the model becomes confused when subtle fibrosis appears in the lung field, leading to missed PF-ILD identification. These examples emphasize the challenges of PF-ILD detection in real-world clinical settings.

### 4.6. Sensitivity to Dataset Size

**Figure 6:**
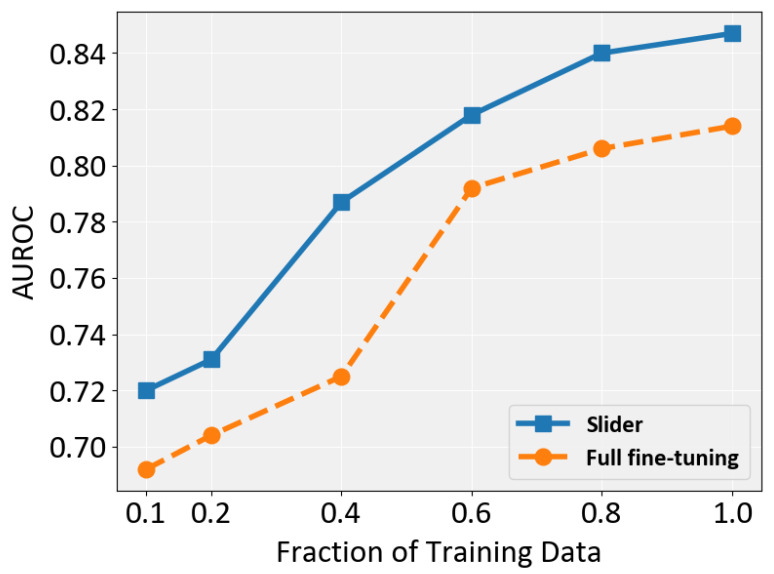
Performance comparison of full fine-tuning and Slider in terms of AUROC across different training data fractions.

To investigate whether Slider is sensitive to the size of the training set, S, we train it on six subsets with different ratios (0.1, 0.2, 0.4, 0.6, 0.8, and 1.0). The results are shown in Figure 6. Overall, Slider consistently outperforms full fine-tuning across all settings, demonstrating its robustness to varying dataset sizes. Both methods benefit from larger training sets, but Slider exhibits a particularly notable improvement when the ratio increases from 0.2 to 0.4. In contrast, full fine-tuning shows optimal performance gain when this ratio changes from 0.4 to 0.6. Importantly, Slider requires only 60% of the training data to surpass full fine-tuning across all dataset sizes, highlighting both its parameter efficiency and its superior data efficiency.

### 4.7. Further Analyses on ILD-Slider

This section further investigates the capabilities of ILD-Slider through a series of experiments.

The dimension on which the Slider should be applied. We set the adapter dimension, *p*, to 192 in Equation (5), which corresponds to a scale factor of δ=4. To systematically investigate the impact of different scale factors δ, we evaluate δ∈{1,2,4,6,8,12} (corresponding to p=768,384,192,128,96,64, respectively). The results are summarized in Table 5.

We found that the best performance is achieved at δ=4, with an AUPRC of 0.790 and an AUROC of 0.847. Moreover, this setting provides the most favorable balance between recall and precision. In contrast, both smaller (δ=2) and larger (δ=12) scale factors yield decreased performance. Notably, a parameter efficiency of δ=4 (3.56 M tunable parameters) outperforms both higher-capacity (δ=1) and lower-capacity settings (δ=12), highlighting an effective trade-off between model capacity and diagnostic accuracy in Slider.

The impact of RGB windowing processing. RGB windowing applies different window levels and widths to emphasize tissue-specific features, enabling the Slider model to capture a richer set of visual cues. Table 6 compares Slider’s performance with and without RGB windowing. Without RGB windowing, its performance markedly drops across most metrics: the AUROC decreases from 0.847 to 0.808, the AUPRC from 0.790 to 0.762, and specificity from 0.840 to 0.446, indicating a sharp increase in false positives. Although recall increases from 0.730 to 0.919 due to the model generating more false positive predictions, this comes at the cost of reduced precision (0.750 to 0.523) and overall accuracy (0.796 to 0.634).

These results demonstrate that RGB windowing yields more balanced and robust diagnostic performance by enhancing the visibility of fibrotic regions while preserving discriminative power for both positive and negative classes in PF-ILD identification.

The impact of using representative slices. We support the use of RSs for PF-ILD identification because they capture anatomically consistent lung parenchyma regions determined by PMs. To evaluate their effectiveness, we compare the proposed Slider model using RSs with randomly selected slices across three runs with different random seeds, as shown in Table 7.

The results indicate a clear and consistent advantage when using RSs. Compared to random slices, RSs improve the AUROC from 0.817 to 0.847 and the AUPRC from 0.739 to 0.790, reflecting stronger overall discrimination and more reliable positive class predictions. Similarly, the F1-Score increases from 0.689 to 0.740. The specificity also increases from 0.744 to 0.840, indicating that RSs help reduce false positives. These improvements demonstrate that PM-guided RS selection not only enhances sensitivity to disease-relevant regions but also minimizes noise from non-informative slices, leading to more accurate and robust PF-ILD identification with Slider.

Effect of kernel shape in Slider. To assess the impact of incorporating slice-level information in Slider for PF-ILD identification, we evaluated different 3D convolution kernel shapes (Table 8). The results show that slice-level modeling plays a crucial role in achieving strong diagnostic performance. When the kernel only captures spatial context without inter-slice information (1×3×3), the AUROC drops to 0.797 and the AUPRC to 0.718, indicating reduced discriminatory ability. Conversely, purely slice-wise kernels (our default setting) without spatial aggregation (3×1×1) achieve the best results, with an AUROC of 0.847 and an AUPRC of 0.790, suggesting that inter-slice context is more critical than additional spatial filtering for PF-ILD identification.

The 3×3×3 kernel, which combines both spatial and slice-level information, yields a competitive AUROC (0.843) but underperforms compared to the 3×1×1 kernal, possibly due to fine-grained slice-level patterns that are relevant to disease progression being over-smoothed. The 1×1×1 kernel, lacking both spatial and slice context, performs worst, confirming that contextual cues, particularly along the slice dimension, are indispensable for PF-ILD identification with Slider.

## 5. Discussion

Clinical translation. ILD-Slider can efficiently identify suspected PF-ILD cases from a limited number of RSs, supporting its prioritization for longitudinal evaluation and further examinations. It has the potential to facilitate screening and reading, particularly in settings with limited access to expert radiologists. However, the final clinical decision should always rely on both integration of longitudinal assessments and clinical findings.

Longitudinal label interpretation. Although the outcome is defined longitudinally, a single-time point HRCT can encode prognostic information. Prior studies [64,65,66] have shown that baseline fibrotic burden and HRCT features are predictive of progression and mortality in fibrosing ILD. We therefore hypothesize that ILD-Slider primarily relies on a fibrosis-weighted parenchymal texture signature, favoring reticulation and traction bronchiectasis over transient or inflammatory ground-glass changes, as well as on apex-to-base (craniocaudal) gradients captured by RSs. These imaging characteristics are commonly observed in fibrosing ILD, particularly when a UIP-like pattern is present (e.g., basal- and subpleural-predominant fibrosis with an apicobasal gradient).

Limitations. In this study, PF-ILD labels are assigned based on clinical diagnostic criteria derived from retrospective medical records, including ≥10% fibrosis, a decline in FVC, and evidence of clinical and radiological progression. Accordingly, estimations made solely from cross-sectional imaging findings are, by definition, subject to an inevitable degree of discrepancy. In particular, longitudinal declines in respiratory function may encompass aspects that are not directly observable through imaging, thereby representing a potential factor that constrains the upper bound of the model’s performance. Furthermore, our dataset was collected from two hospitals within the same geographic region in Japan. It may limit the generalizability of ILD-Slider to other populations, scanners, or clinical settings.

Future work. To overcome the inherent limitations of imaging alone, future work should explore multimodal modeling that incorporates pulmonary function metrics, such as FVC, clinical symptoms, and biomarkers, or directly train models on disease progression using longitudinal data. In addition, important next steps in this field include external calibration and validation, decision curve analysis to evaluate clinical utility, and the development of radiologist-facing user interfaces with enhanced explainability.

## 6. Conclusions

We propose a new method, ILD-Slider, which is a parameter-efficient and lightweight framework for PF-ILD identification from a limited number of CT slices. By introducing anatomy-based PMs and a PSM strategy, ILD-Slider effectively selects RSs, substantially reducing computational cost while preserving diagnostic accuracy. Extensive experiments on datasets from two independent medical facilities demonstrated that ILD-Slider achieves robust performance (an AUPRC of 0.790 and an AUROC of 0.847 with only five RSs), confirming its practicality for real-world clinical settings. Furthermore, our analysis highlights the benefits of RGB windowing processing, cross-domain generalization, and the feasibility of diagnosing PF-ILD from non-contiguous slices, representing an important step toward broader applicability on public datasets. These findings underscore ILD-Slider as both a data-efficient and computationally efficient solution for early PF-ILD identification.

## Figures and Tables

**Table 1:** Number of non-PF-ILD and PF-ILD cases from the two facilities. UOH and OTMC denote The University of Osaka Hospital (UOH) and the National Hospital Organization Osaka Toneyama Medical Center (OTMC), respectively.

Facility	Non-PF-ILD	PF-ILD	Total
UOH	236	167	403
OTMC	130	80	210

**Table 2:** The results of the PSM-based PM extractor with 1-Up-Down accuracy. The best scores for each PM are highlighted in **bold**.

Model	Upper PM (%)	Middle PM (%)	Lower PM (%)	Avg. (%)
ResNet-101	98.33	96.67	95.0	96.67
DenseNet-169	**100**	**98.33**	95.0	97.78
EfficientNet-b4	**100**	**98.33**	**96.67**	**98.33**

**Table 3:** Performance of Slider on the PF-ILD datasets across different metrics for 3-RS, 5-RS, and 9-RS settings. #Params denotes the number of tunable parameters. Rows shaded in light blue indicate our method. The best score for each setting is shown in **bold**.

Method	RSs	#Params (M)	Acc.	Rec.	Prec.	Spec.	F1	AUPRC	AUROC
Full Fine-tuning	×3	86.58	0.720	0.757	0.622	0.696	0.683	0.692	0.797
Partial Fine-tuning	×3	7.09	0.742	0.811	0.638	0.696	0.714	**0.738**	0.810
Linear Probe	×3	0.0015	0.602	**0.946**	0.500	0.375	0.654	0.726	0.780
Slider	×3	3.56	**0.785**	0.703	**0.743**	**0.839**	**0.722**	0.735	**0.813**
Full Fine-tuning	×5	86.58	0.656	**0.838**	0.544	0.536	0.660	0.782	0.814
Partial Fine-tuning	×5	7.09	0.688	**0.838**	0.574	0.589	0.681	0.760	0.832
Linear Probe	×5	0.0015	0.785	0.730	0.730	0.821	0.730	0.724	0.774
Slider	×5	3.56	**0.796**	0.730	**0.750**	**0.840**	**0.740**	**0.790**	**0.847**
Full Fine-tuning	×9	86.58	**0.763**	0.541	**0.800**	0.911	0.645	**0.771**	0.808
Partial Fine-tuning	×9	7.09	**0.763**	**0.811**	0.667	0.732	**0.732**	0.712	**0.823**
Linear Probe	×9	0.0015	0.731	0.432	**0.800**	**0.929**	0.561	0.718	0.768
Slider	×9	3.56	0.731	0.703	0.650	0.750	0.675	0.751	0.821

**Table 4:** Cross-domain evaluation of Slider on AUROC. Each row corresponds to the facility used for training, and each column corresponds to the facility used for testing.

Domain	UOH	OTMC	UOH and OTMC
UOH	0.750	0.896	0.791
OTMC	0.759	0.921	0.803
UOH and OTMC	0.823	0.875	0.847

**Table 5:** The performance of Slider for different scale factors, δ. The row shaded in light blue indicates our default settings.

Setting	#Params (M)	Acc.	Rec.	Prec.	Spec.	F1	AUPRC	AUROC
δ=1	14.21	0.710	0.622	0.639	0.768	0.630	0.630	0.811
δ=2	7.11	0.720	0.730	0.628	0.714	0.675	0.723	0.806
δ=4	3.56	0.796	0.730	0.750	0.840	0.740	0.790	0.847
δ=6	2.38	0.710	0.730	0.614	0.696	0.667	0.742	0.797
δ=8	1.79	0.774	0.730	0.711	0.804	0.720	0.777	0.816
δ=12	1.19	0.785	0.649	0.774	0.875	0.706	0.770	0.837

**Table 6:** The performance of Slider with (w/) and without (w/o) RGB windowing processing. The row shaded in light blue indicates our default settings.

Setting	Acc.	Rec.	Prec.	Spec.	F1	AUPRC	AUROC
w/o RGB	0.634	0.919	0.523	0.446	0.667	0.762	0.808
w/RGB	0.796	0.730	0.750	0.840	0.740	0.790	0.847

**Table 7:** The performance of Slider on randomly selected slices (mean ± std over three runs with different random seeds) and RSs. The row shaded in light blue indicates our method.

Method	Acc.	Rec.	Prec.	Spec.	F1	AUPRC	AUROC
Random	0.738±0.05	0.730±0.09	0.659±0.08	0.744±0.08	0.689±0.06	0.739±0.03	0.817±0.02
Slider	0.796	0.730	0.750	0.840	0.740	0.790	0.847

**Table 8:** The performance of Slider on different kernel shape settings. The row shaded in light blue indicates our default settings.

Kerneal Shape	Acc.	Rec.	Prec.	Spec.	F1	AUPRC	AUROC
1×1×1	0.591	0.919	0.493	0.375	0.642	0.784	0.809
1×3×3	0.753	0.568	0.750	0.875	0.646	0.718	0.797
3×3×3	0.763	0.811	0.667	0.732	0.732	0.749	0.843
3×1×1	0.796	0.730	0.750	0.840	0.740	0.790	0.847

## Data Availability

The data presented in this study are available on request from the corresponding authore due to privacy restrictions.
